# Supplementary material for: The Clinical Characteristics and Prognostic Factors of Primary Extramammary Paget’s Disease Treated with Surgery in Anogenital Regions: A Large Population Study from the SEER Database and Our Centre
Source: J Clin Med. 2023 Jan 11;12(2):582. doi: 10.3390/jcm12020582 (PMC9867386; doi:10.3390/jcm12020582)
Supplement: Supplementary file 1 [file jcm-12-00582-s001.zip › jcm-2153044-supplementary.pdf]

**Table S1.** SEER baseline

| Variables | Overall |
|---|---|
| N | 688 |
| Age (mean (SD)) | 69.68 (11.36) |
| Age65 (%) | |
| <65 | 237 (34.45) |
| >65 | 451 (65.55) |
| Sex (%) | |
| Female | 502 (72.97) |
| Male | 186 (27.03) |
| Year2005 (%) | |
| <2005 | 318 (46.22) |
| >2005 | 370 (53.78) |
| Races (%) | |
| American Indian/Alaska Native | 478 (69.48) |
| Asianor Pacific Islander | 4 (0.58) |
| Black | 199 (28.92) |
| Unknown | 3 (0.44) |
| White | 4 (0.58) |
| SEER stage (%) | |
| Localized | 542 (78.78) |
| Regional | 110 (15.99) |
| Distant | 11 (1.60) |
| NA | 25 (3.63) |
| Status of lymph nodes | |
| N+ | 17 (2.47) |
| NA | 671 (97.53) |
| Diameter (cm) | |
| 0-5 | 231 (33.58) |
| 5-10 | 53 (7.70) |

| | |
|---|---|
| >10 | 14 (2.03) |
| NA | 390 (56.69) |
| Hispanic | |
| Non-Spanish-Hispanic-Latino | 635 (92.30) |
| Spanish-Hispanic-Latino | 53 (7.70) |
| Sites of EMPD (%) | |
| Anus | 22 (3.20) |
| Penis | 20 (2.91) |
| Scrotum | 151 (21.95) |
| Vagina | 3 (0.44) |
| Vulva | 492 (71.51) |

**Table S2.** EMPD baseline

| Variables | Overall |
|---|---|
| N | 176 |
| Age (mean (SD)) | 65.81 (9.57) |
| Age (%) | |
| <65 | 79 (44.89) |
| >65 | 97 (55.11) |
| BMI (mean (SD)) | 24.41 (3.17) |
| BMI (%) | |
| <25 | 103 (58.52) |
| >25 | 73 (41.48) |
| Sex (%) | |
| Male | 157 (89.20) |
| Female | 19 (10.80) |
| ASA | |
| 1-2 | 152 (86.36) |
| 3-4 | 24 (13.64) |
| Comorbidity (%) | |
| No | 78 (44.32) |
| Yes | 98 (55.68) |
| SEER stage (%) | |
| Localized | 134 (76.14) |
| Regional | 40 (22.73) |
| Distant | 2 (1.14) |
| Sites of EMPD (%) | |
| Vulva | 19 (10.80) |
| Penis | 16 (9.09) |
| Scrotum | 141 (80.11) |
| Diameter (mean (SD)) | 6.41 (2.91) |
| Diameter (cm) | |
| <5 | 81 (46.02) |
| 5-10 | 80 (45.45) |
| >10 | 14 (7.95) |
| NA | 1 (0.57) |
| Margin (%) | |
| Negative | 144 (81.82) |
| Positive | 31 (17.61) |
| NA | 1 (0.57) |
| Lymphadenectomy(%) | |
| No | 168 (95.45) |

| | |
|---|---|
| Yes | 8 (4.55) |
| Status of lymph nodes | |
| N+ | 6 (3.41) |
| NA | 170 (96.59) |
| Invasion level (%) | |
| Level1 | 133 (75.57) |
| Level2 | 12 (6.82) |
| Level3 | 31 (17.61) |
| LVI (%) | |
| No | 172 (97.73) |
| Yes | 4 (2.27) |
| Chemotherapy (%) | |
| No | 171 (97.16) |
| Yes | 5 (2.84) |
| Radiation (%) | |
| No | 105 (59.66) |
| Yes | 71 (40.34) |
| Reconstruction (%) | |
| Direct closure | 67 (38.07) |
| Free skin flaps | 24 (13.64) |
| Skin grafting | 85 (48.30) |
| Surgical type (%) | |
| WLE | 44 (25.00) |
| MMS | 132 (75.00) |
| Intervals | |
| <2 year | 65 (36.93) |
| >2 year | 111 (63.07) |
